# Cancer of the Cervix Uteri

**Published:** 1881-05

**Authors:** John G. Blake


### CANCER OF THE CERVIX UTERI.

By Dr. John G. Blake.

Malignant disease of the cervix uteri is a malady often coming under the notice of the hospital physicians and gynæcologist. The distressing nature of the disease, the continuous pain and suffering, the duration, and the inevitable result, all appeal powerfully to our sympathies, and our professional pride is aroused to lessen, if we cannot cure, the distress it occasions. Unfortunately for both physician and patient, it often happens that the disease is not recognized or advice sought until too late. We are all familiar with the errors of patients when undertaking to diagnosticate their own diseases, but it has been my lot to find more than the usual number of mistakes on this subject. Unfortunately this ignorance is not by any means confined exclusively to the patient, for within a short time I have met in private practice two cases of advanced cancer of the womb in which neither examination nor diagnosis had been made, and in consequence no attempt at treatment. One of these cases died within a few weeks, the disease being already far advanced. Though experience of this kind is uufortunately too common, I must, in justice to the younger members of the profession, say that they would hardly be open to the charge of ignorance or carelessness. Twenty-four years ago the opportunities for gynæcological study did not exist in Boston for medical students. I remember the only opportunity I had for viewing the cervix was occasionally when making a visit with Dr. Minot, to whom I am glad to make personal acknowledgement for his interest and kindness to the class in those days. Matters have improved a great deal since, and no excuse of this kind can any longer be alleged by the faithful student as a reason for want of familiarity with uterine lesions. At both of the large hospitals clinical instruction and demonstration are given by the medical staff throughout the year; dispensary clinics also exist, and special instructors teach the sujbect thoroughly during the last year or years of study. All this awakening of interest has stimulated inquiry, and the result has been a vast advance in knowledge, both of diseases to which this organ is subject and of their successful treatment. As Americans we may feel a pardonable pride that our countrymen are among the foremost of the world in this branch of medicine. Judging by the men at present engaged in this work, and their successful practice, we shall continue in the fromt rank. All this is simply introductory to the reports of an attempt to relieve certain cases of malignant disease of the neck of the womb, which attempts were based upon reports of similar cases treated successfully, and which in one case were helped by the personal advice and counsel of Dr Marion Sims, to whom American gynæcology is so deeply indebted.

*Cancer of Prolapsed Uterus. Case I*—January 3, 1880. E. B., forty two, widow, born in Ireland; catamenia first appeared at the age of fourteen, were regular and painless, usually lasting about a week, but occasionally being abbreviated to three days. Was married when twenty-two; has had no children or miscarriages, but soon after marriage the flow became excessive, lasting two weeks, in the intervals the patient suffered from severe leucorrhœa until twelve or thirteen years ago, when it ceased. Says that coition was never painful, and can give no reason for her sterility. A little while after marriage began to complain of pains in back, side, and lower part of abdomen; pains were not very severe. Says her uterus came down about two years after marriage, so that it became visible. She treated it with a T bandage, and was comparatively comfortable until six months ago. A year ago the menstrual discharge began to diminish, and now it is very scanty indeed. Six months ago first had frequent and painful micturition with blood in the urine at times.

Appetite poor, sleeps badly; bowels always costive, being moved only by medicines. Temperature and pulse normal. No œdema or cough.

On examination find complete prolapse of vagina, terminating in a large conical shaped mass, with base outwards, measuring about five inches in diameter, of ulcerated surface, discharging pus, and bleeding from slight friction. Examination per rectum and suprapubic reveals absence of uterus in normal position. Examination by sound confirms the same.

Upon microscopical examination Dr. E. G. Cutler pronounced it cancer. Following is the result of a similar examination by Dr. R. H. Fitz: "Find your specimen covered with epithelium, resembling that from cervix rather than fundus The tissues beneath are largely fibrous, with abundant round cells infiltrated. Still deeper are fusiform cells of muscular tissue. The tissues appeared to be continuous from the surface downwards. The examination would suggest prolapse or inversion rather than polypus, although it would be difficult to establish a diagnosis from the microscopical examination without knowing more about the relations of the specimen."

January 13th Complains somewhat of pain, relieved by morph. sulph., p. r. n ℞ vin. pepsin., tr. gentian. comp., aa ℥ss. t i. d.

January 25th. Dr. J. Marion Sims saw the patient in consultation with Drs. Lyman and Blake, and strongly advised removal of the diseased mass, including uterus and its appendages.

January 28th. Operation by Dr. Blake. Patient being under ether, a circular incision was made sufficiently high to avoid diseased tissue. After careful dissection vagina was cut through, and the uterus, ovaries, broad ligaments, and Fallopian tubes were carefully isolated from surrounding tissue, special pains being taken to avoid injury to rectum or bladder. Dissection in relation to latter organ was made with special care to avoid injury to urethra, base of bladder, and ureters. After uterus and appendages were well drawn forward, a ligature was passed above fundus, tied very tightly, and parts below, comprising the uterus, ovaries, round ligaments, and Fallopian tubes, were separated and removed. The operation was necessarily somewhat protracted, occupying three-quarters of an hour. On account of vascularity of diseased tissue considerable hæmorrhage was unavoidable.

Patient sank a good deal during operation and rallied but feebly in response to stimulants. Efforts to promote reaction during the rest of the day were unsuccessful; patient gradually sank, and died at 9:50 P M., about twelve hours after the operation.

Examination of whole specimen by Dr. E G. Cutler. Greater portion of tumor was composed of cervix and vagina. The wall was infiltrated with a new growth, cancerous, which extended up into the fundus, evident to senses of both touch and sight. The microscopical examination showed it to be of the epithelial variety, and it did not seem to have invaded glands on the broad ligament. The peritoneal surface was the seat of old inflammation, and there were numerous adhesions, old and new, in Douglas's space, between the uterus and the prolapsed portion of vagina. Left ovary was in a state of cystic degeneration. Left Fallopian tube was accluded, and contained some pus. Right tube and ovary were apparently healthy.

*Case II.* March 1. S. M., fifty-five, and married; was well until two or three months ago, though previous to this time had lost some flesh and strength, and had been troubled with vague pains in back, etc., when she was attacked with flowing, coming on gradual at first, but very severe at present.

Was married at eighteen, and has had eleven children at full term, and no miscarriages. About this time first noticed a tumor in the upper part of the vagina, which has rapidly increased in size, and is now the cause of much local trouble and pain. States that any sudden exertion will cause an immediate flowing. Bowels constipated. Micturition difficult. Tongue clean. Anorexia. Pulse and temperature normal.

Examination per vaginam brings finger in contact with a large lobulated mass of diseased tissue, completely encircling and growing fr m cervix. Anteriorly disease presents a globular appearance, about the size of a hen's egg. Posteriorly, disease irregular in shape, and filling pretty well upper vagina. Disease is friable to touch, portions of mass disintegrating, and coming away as result of digital examination. Appearances are identical with those found in advanced malignant disease of the cervix.

Operation. In consultation with Dr. Lyman it was decided to remove as much of diseased tissue as possible. Patient declined being etherized. Uterus having been drawn well down it was found that the ecraseur would most effectually remove the disease. Wire was passed around mass, and tension applied when the wire broke. Chain was then substituted, and diseased portion including a small secretion from cervix, removed without injury either to urethra or bladder. A small mass remaining on posterior wall was thoroughly removed with Lieman's scoop, and entire stump freely touched with strong nitric acid, afterwards an alkaline solution was applied, followed by a glycerine tampon. Morph. sulph., one-sixth grain, subcutaneously.

March 3d. Second dressing made. Nitric acid applied, followed by glycerine tampon and dry cotton. Hot vaginal douche every morning. Dr. Cutler examined a specimen of the tissue, and pronouned it cancer.

March 9th. Incised surface covered with healthy granulations. ℞ Ferrated tr. cinchona, ʒi. t. i. d. ℞ Fowler's solution, gtt. v., t i. d.

March 27th. Patient has had applications of nitric acid once in five days, followed by glycerine dressing. Granulating surface now contracted to size of a quarter dollar, of healthy appearance, and without discharge. General condition good.

April 2d. A growth of exuberant granulations on anterior lip (cancer ?) was scooped off, and nitric acid applied, followed by glycerine tampon. General condition improving.

April 9th. Disease involves whole posterior lip, having extended with marked rapidity; cancerous nodules over incised surface of anterior lip.

April 12th. Diseased mass on posterior lip was removed with knife, when an opening was made into the peritonæum, admitting tip of finger. One silver suture inserted. Morph. sulph., p. r. n.

April 14th. Complains of occasional darting pains in abdomen.

April 15th. No febrile disturbance.

April 23d. Diseased surface looks well, and shows only a small granulating part. Suture removed and parts united. Patient was discharged relieved; to be examined once a month.

August 23d. After leaving hospital patient was very well for two months, when the pain returned; she kept about her work until three weeks ago, when the flowing again began. General health in the meantime has been good. Examination per vaginum shows a mass of granulations growing from posterior lip, extending up cervical canal, and beginning to invade upper vaginal wall. Mass thoroughly removed with curette, scoop and scissors, and saturated solution of chromic acid applied. ℞ Morph. sulph., p. r. n

August 24th. Parts looking very well. Chromic acid again applied

August 26th. Glycerine tampon applied to cervix twice a day. ℞ Ferri et quinæ cit., gr. v., vin Xerici, ℥ss. t. i. d.

September 6th. Slough almost entirely thrown off. Parts look much more healthy.

September 11th. Discharged, relieved.

*Case III.*—March 10th. C. D., forty and widow. Has had four children, the last one thirteen years ago. No miscarriages. Catamenia regular until last Christmas, when she first noticed a discharge from the vagina, with a very offensive odor, which was followed by flowing two days after, this being her regular time to be "unwell." This sort of thing continued, the flowing being much increased by any heavy work, together with pains in back, especially on left side, anorexia, constipation, etc. Micturition normal. Tongue furred. Temperature 100° F. Pulse 115.

March 18th. Examination per vaginum showed whole of both posterior and anterior lips, as well as cervix to within a quarter inch of vaginal junction, one mass of ulcerating growth, which bled freely when manipulated, the presenting surface being two inches in diameter. Vagina tamponed with Monsel's solution on cotton. ℞ Ferri et quiniæ cit., gr. v., vin. Xerici, ℥ss. t. i. d.

March 19th. Patient etherized. Examination showed the whole cervix to consist of one mass of epithelioma, extending under mucous membrane beyond vaginal junction. Diseased part removed up to vaginal junction by means of curette, and as far as internal os. Hæmorrhage not excessive. Nitric acid applied, and cervix tamponed with cotton soaked in Monsel's solution.

March 20th. Hot carbolized vaginal douches twice a day.

March 30th. Once in three days surface has been dressed with a nitric acid and glycerine tampon. Parts have contracted one half, but whole surface shows a sloughing condition, with here and there a prominent cancerous nodule. Free discharges of pus noticed at the time of each dressing.

April 5th. Posterior vaginal wall at junction of cervix consists entirely of cancerous mass easily breaking down; same condition anteriorly. Mass removed by curette and scissors, even on to vaginal wall. Nitric acid employed, followed by dressing of Monsel's solution.

April 9th. Dressing removed, and several small cancerous nodules on posterior and one on anterior wall removed. Nitric acid applied, followed by glycerine tampon.

May 11th. Growth extending very rapidly, occupying surfaces previously denuded by operations, also involving body of uterus through the inner os and both walls of the vagina, so that any attempt to remove the growth would open into the peritoneal cavity.

General condition greatly improved. Discharged, relieved, at own request.

*Case IV.*—December 12, 1878. A. N., widow, aged thirty-seven, enjoyed good health until two years ago, when catamenia became irregular. Has had three children at full term, the youngest being nine years old. For the past two weeks has been flowing steadily; no offensive discharge from vagina. Appetite poor; bowels constipated. Pulse and temperature normal.

December 13th. Examination per vaginam shows the whole cavity of the cervix to be ulcerated and eroded, admitting tip of finger as far as internal os. Vaginal aspect healthy. Thin sanguineous pus exuding from cervix. Plug of cotton soaked in glacial acetic acid inserted into cervix, and glycerine tampon applied.

December 16th. Cervix secretes pus, comparatively healthy in appearance. Parts washed with carbolic solution, and ulcerations touched with glacial acetic acid; plug of lint soaked in the acid inserted into the cervix.

December 20th. Carbolized injections, with slippery-elm tea, night and morning.

December 22d. Cervix touched with glacial acetic acid. No pain; general condition good.

January 1st. ℞. Ferri et quiniæ cit. gr. v. t. i. d. Friedrichshall before breakfast.

January 7th. Glycerine tampons inserted twice per day. Os looking well. Lunar caustic applied, followed by glycerine tampon. Suppository of morphia and belladonna at night.

January 29th. Patient has been made comfortable with morphia. General condition good. Examination per vaginam shows complete cicatrization of cervical tissue. Uterus moderately retroflexed and movable. Cicatricial tissue indurated, due to operations. A sero-purulent leucorrhœa, probably of vaginal origin.

January 31st. Discharged, relieved.

*Case V.*—Mary P., married, age forty-three, entered the hospital January 27th, with the following history: Has had ten children; labors normal; oldest child twentytwo, youngest three years of age. Menstruates regularly every four weeks: flows but three days; uses but two or three napkins each day. Has had more or less uterine trouble for the past year. For the past three months has had severe pain in the back and in the lower part of abdomen. An abundant watery, offensive discharge from the vagina. For the past week has noticed a swelling in the lower part of abdomen on the right side. Difficult micturition for the past two months. Bowels constipated. Appetite good. An examination showed cervix much destroyed from some previous caustic application. Os open, admitting the finger freely to interior of uterine cavity. Interior of uterus found to consist of softened, somewhat pulpy, nodulated tissue, which bleeds freely, and is evidently of a malignant character. Much pale, flabby, exuberant granulations around what remains of the cervix. Uterus not much enlarged. External examination discovers a mass somewhat indistinct in right ovarian region. A portion was removed from inside uterus and sent to Dr. Cutler, who pronounced it to be epithelioma. Patient was ordered citr. quinine and iron, gr. v. t. i. d. Fowler's sol., five minims after each meal. Hot carbolized douches twice a day.

February 3d. Interior of uterus thoroughly curetted and all fungoid tissue removed, after which a strong solution of chromic acid was applied to curetted surface. Retention of urine followed the operation, but lasted only two days. Ordered opium p. r. n.

February 10th. Pain in back quite severe. Discharge from vagina much less offensive. Douches come away quite clear.

From February 15th there was continued improvement in both general and local condition. Expressed herself as feeling well, except that she has a pain in small of back.

March 2d. On examination no apparent evidence of disease was found.

March 5th. Discharged, relieved.

*Remarks.*—These cases fairly represent diseases of this organ as it usually comes under the physician's care, and the question to decide is whether surgical interference is justified by the results. While at first but little can be said in favor of operation from the account of these results, yet we must remember that temporary relief from pain and the prolongation of life for months, if not years, are considerations which control our decisions in other cases and which are entitled to great weight here. The unfortunate termination of the first case was, in a great degree, anticipated; yet I cannot but feel that the operation was called for. I may say that whatever doubts lingered in my mind was removed by Dr. Sims, who kindly saw the patient, and strongly advised extirpation as performed. Operations of such severity are naturally attended by great shock to the nervous system as well as much loss of blood, a condition dependent on the nature of the disease and length of time required. Besides, the rarity of operations of this character leaves a want of skill which is not easily remedied. I suppose I only voice the sentiment of every gentleman present in saying that in future operations many points will be treated differently, and the chances of cure increased by experience gained in each new case. The records of ovariotomy give abundant proof of this.

In relation to Case II. the result has been very different. This patient continues quite well in general health, able to attend to her household duties, free from pain and hæmorrhage, and suffering only from a leucorrhœal discharge, which disinfectant injections render inoffensive. There is as yet no cancerous cachexia or vesical irritation, and from present appearances her prospect of life appears good for many months at least. This result has been obtained only by constant supervision and removal of the disease on its reappearance. As often as once a month either the curette has scraped away exuberant granular adhesions, or applications of nitric or chromic acid have kept the disease in subjection. As yet there is no evidence of the fundus uteri becoming invaded, though it is only a matter of time when both that and the pelvic viscera will become involved.

Case III., operated upon by Dr. Doe and kindly permitted by him to become one of this group, has, I understand, not done as well, from the fact that circumstances did not allow him to follow up the treatment so persisently. He will give us full details of the subsequent history.

Case IV. was primarily under the care of the late Dr. George H. Gay, and afterwards of our president, Dr. Lyman, who had the opportunity of treating it in its active stage. From him it descended to me as his successor in service at the hospital. At that time very little in the shape of active treatment remained to be attended to, and it is briefly alluded to here so that he may give us an accurate account of the earlier stages of the disease and treatment, while I can testify to the cure and entire absence of disease up to the present time. I recently carefully examined the patient at my office, in order to be able to report her condition one year after leaving the hospital.

Two of these cases strikingly resemble a group treated somewhat similarly by Dr. Marion Sims, and reported and commented on by him in the *American Journal of Obstetrics.* As the subject is doubtless familiar to members of this Society I have refrained from making reflections or suggestions, preferring to offer the paper as a text upon which members can express their opinions fully. I am for myself decidedly in favor of surgical interference and complete removal of the disease at the earliest possible opportunity.

I am equally decided in my opinion that the disease is primarily local in origin, often starts from a lacerated cervix or long-continued abrasion, erosion, or so called ulceration of the os, and that if we could prevent solutions of continuity of the cervix uteri, or cure them before they become chronic, we should have fewer cases of malignant disease of that organ to treat.

